# Whence the birds: 200 years of dinosaurs, avian antecedents

**DOI:** 10.1098/rsbl.2024.0500

**Published:** 2025-01-22

**Authors:** Daniel J. Field, M. Grace Burton, Juan Benito, Olivia Plateau, Guillermo Navalón

**Affiliations:** ^1^ Department of Earth Sciences, University of Cambridge, Cambridge, UK; ^2^ Museum of Zoology, University of Cambridge, Cambridge, UK

**Keywords:** skeletal pneumaticity, flight apparatus, brain evolution, palate, bird evolution, dinosaur evolution

## Abstract

Among the most revolutionary insights emerging from 200 years of research on dinosaurs is that the clade Dinosauria is represented by approximately 11 000 living species of birds. Although the origin of birds among dinosaurs has been reviewed extensively, recent years have witnessed tremendous progress in our understanding of the deep evolutionary origins of numerous distinctive avian anatomical systems. These advances have been enabled by exciting new fossil discoveries, leading to an ever-expanding phylogenetic framework with which to pinpoint the origins of characteristic avian features. The present review focuses on four notable avian systems whose Mesozoic evolutionary history has been greatly clarified by recent discoveries: brain, kinetic palate, pectoral girdle and postcranial skeletal pneumaticity.

## 1. Introduction

The year 2024 marks the 200th anniversary of *Megalosaurus*, the first non-avian dinosaur to be validly named [1]. Though not initially recognized as such, that discovery can be viewed as the genesis of humanity’s enduring fascination with the clade Dinosauria, a term first coined in 1842 [2]. While 200 years of research on dinosaurs has revealed a huge amount about the extinct diversity of the Mesozoic Era, arguably the most important single insight arising from the vast body of dinosaur research is the revelation that Dinosauria includes all birds, living and extinct. This insight arose gradually (e.g. [3–12]), yet today the emergence of birds among dinosaurs stands as one of the best understood macroevolutionary transformations in the entire history of life, and investigations of the evolutionary origins of the distinctive ‘avian’ characteristics that set birds apart from other reptiles remains a vibrant area of palaeontological research. A rush of discoveries beginning in the early 1990s led to fundamental new insights regarding the evolutionary origins of many aspects of avian biology, which have been summarized in numerous well-cited reviews published between the late 1990s and 2010s (e.g. [13–17]). This extraordinary pace of discovery continues unabated, and the years since have seen a wealth of new data emerge that further clarify the evolutionary origins of quintessentially ‘avian’ features.

Crown group birds arose towards the end of the Cretaceous Period [18–21]. The earliest crown birds would have lived alongside representatives of all three major dinosaurian subclades (Ornithischia, Sauropodomorpha and Theropoda) as well as many lineages of non-neornithine avialans until the end-Cretaceous mass extinction event left crown birds as the only surviving representatives of Dinosauria [22–24]. The rich Mesozoic fossil record of stem-group birds (including pterosaurs, non-avian dinosaurs and non-neornithine avialans) is the place to turn for insights into the evolutionary origins of crown bird characteristics. In the present review, we aim to summarize recent advances clarifying the origins of several specialized aspects of crown bird anatomy underpinning distinctive avian functional capabilities. We focus on discoveries emerging in recent years (i.e. since 2018) regarding the evolution of the following key traits: (i) an enlarged and anatomically modern brain (enabling advanced cognition and behavioural complexity), (ii) a kinetic palate (facilitating cranial kinesis), (iii) a geometrically modern pectoral girdle (underpinning sophisticated powered flight) and (iv) postcranial skeletal pneumaticity (PSP; related to lightening the skeleton). Feathers, one of the hallmarks of bird morphology and a textbook example of a ‘crown bird feature’ observable in Mesozoic dinosaurs [25–28], are treated in a separate review in this volume. We also identify enduring knowledge gaps that limit a comprehensive understanding of these transitions in the hope that these will be filled in the coming years.

### (a) Brain evolution: thinking like a bird

**Figure 1.**
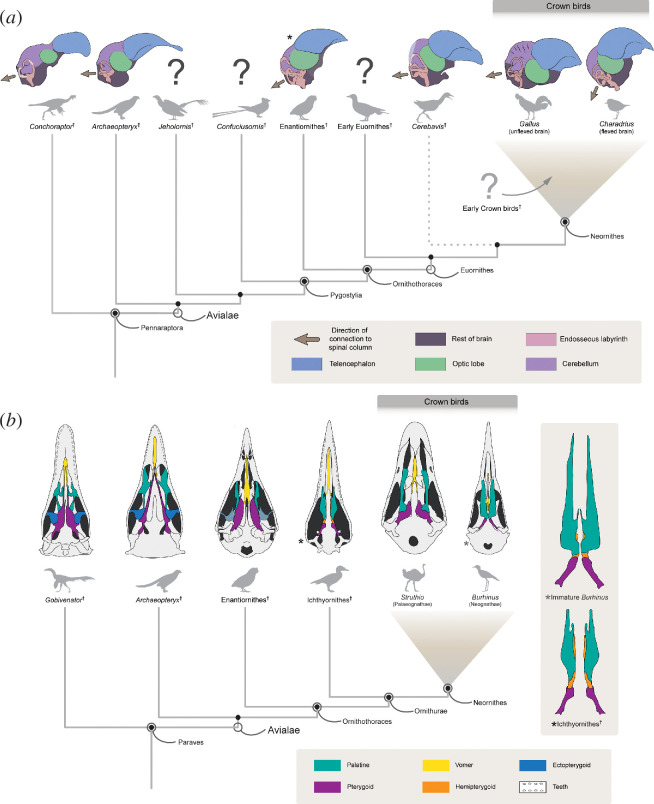
Evolution of the avian central nervous system and bony palate. (*a*) *Navaornis* (starred) illustrates avialan brain and inner ear morphology in an enantiornithine; however, endocranial morphology of non-neornithine avialans phylogenetically stemward and crownward of Enantiornithes remains poorly known. (*b*) Left: paravian skulls in ventral view (modified from [35–38]) with palate osteology highlighted. Light blue ectopterygoids reflect uncertain presence in Enantiornithes. Stars next to the skulls of Ichthyornithes and *Burhinus* correspond to the enlarged palates illustrated to the right of the phylogeny. Right: morphological comparison of the pterygoid–palatine complex in an immature neognath (*Burhinus capensis*; above) and Ichthyornithes (below; composite reconstruction of *Janavis* and *Ichthyornis*, modified from [37] and [39]).

Among living vertebrates, only birds rival mammals in terms of relative brain size and behavioural complexity, and the exceptional problem-solving capabilities and flamboyant displays of many extant birds are well studied [29]. Relatedly, the morphology of the avian central nervous system is highly divergent with respect to all other known reptiles, including non-avian dinosaurs. For instance, the brain of crown birds is larger and more globular than that of other reptiles due to the volumetric expansion of key brain regions, particularly the telencephalon (cerebrum) and the cerebellum [30–33]. In many crown birds the caudal portion of the brain flexes ventrally, which re-orients the spatial relationships of its main anatomical subdivisions (e.g. the telencephalon sits dorsal to the optic lobes) and redirects the connection with the spinal column ventrally rather than caudally as in most non-avian reptiles [34] (figure 1*a*). This derived neuroanatomy co-occurs with similarly derived morphologies of the inner ear (e.g. an enlarged and sinusoidal anterior semicircular canal [40,41]). These morphological transformations of the brain and associated sensory structures have traditionally been linked to the origin of avian flight (e.g. [33,34]) and other adaptations at least partially underpinning avian evolutionary success (e.g. survivorship through the Cretaceous–Palaeogene (K–Pg) mass extinction; [39]).

Tight anatomical correspondence between the bony endocranial cavities and underlying central nervous system enable reconstructions of endocranial morphology in Mesozoic fossils illuminating the evolutionary history of distinctly ‘avian’ neuroanatomical specializations [42,43]. This has enabled the reconstruction of brain morphology in *Archaeopteryx* [44], which is similar overall to that of closely related non-avian maniraptorans in exhibiting limited expansion of the telencephalon, cerebellum and optic lobes, as well as a similarly unflexed brain connecting to the spinal cord caudally [30,44].

Nonetheless, the rarity of complete, three-dimensionally preserved fossils from the Mesozoic Era has long obfuscated the evolutionary transition from the plesiomorphic central nervous system of the earliest avialans like *Archaeopteryx* to the derived neuroanatomy of crown birds (figure 1*a*). This leaves a gap of more than 70 Ma in which our understanding of the evolution of the modern avian central nervous system is practically non-existent (figure 1*a*), an interval that witnessed the origination of all major groups of Mesozoic avialans, as well as the earliest phylogenetic divergences among crown birds.

Among non-neornithine avialans, the clade Enantiornithes occupies a phylogenetically pivotal position as the sister group to Euornithes, which includes crown birds (figure 3). Enantiornithes dominated Mesozoic avifaunas globally and represents the first great radiation of bird-like animals [45], yet despite their abundance and diversity, the first detailed insights into enantiornithine endocranial morphology have only recently emerged ([36,46],). The recently described enantiornithine *Navaornis hestiae* is represented by unusually immaculate, three-dimensionally preserved remains, enabling a nearly complete reconstruction of its skull and endocranial morphology ([36]). Although its skull geometry is remarkably like that of crown birds, its endocranial morphology retains numerous plesiomorphic features, such that it is roughly intermediate between *Archaeopteryx* and crown birds (figure 1*a*), suggesting a notable degree of independence with respect to evolutionary changes in the skull and brain. These plesiomorphies include a small cerebellum relative to the rest of the brain, and a telencephalon with a mediolateral expansion exceeding the degree observed in *Archaeopteryx* [30,44] and known non-avian pennaraptoran dinosaurs [47,48] (figure 1*a*), but less than that observed in most crown birds and crownward avialans in which the telencephalic hemispheres envelop the dorsal surface of the optic lobes [43,49] (figure 1*a*). Despite these plesiomorphic attributes, *Navaornis* also exhibits several neuroanatomical features only previously known from crown birds and the crownward-most stem birds such as large optic lobes positioned ventral to the telencephalon, and a bony inner ear labyrinth comparable in shape, but much larger, than that of many crown birds. Although the flight apparatus of enantiornithines may have been broadly functionally comparable with that of crown birds, the lack of a full suite of crown-like neuroanatomical features in the brain of *Navaornis* casts doubt on a tight association between the origin of powered flight and modern avian neuroanatomy.

Recent work provides the clearest insights yet into the neuroanatomy of the ancestral crown bird [50], illustrating that ‘modern type’ avian brains were present among the earliest known stem palaeognaths. However, phylogenetically pinpointing the evolutionary origin of the modern-type avian brain remains challenging. The earliest apparent evidence of modern-type avian brain morphology comes from a single, isolated 100-million-year-old fossil braincase [49,51]. Unfortunately, the phylogenetic affinities of this fossil (*Cerebavis cenomanica*) remain enigmatic due to the absence of associated skeletal remains. Moreover, taphonomic deformation (e.g. [39,52]) and fossil incompleteness (e.g. [53]) continue to hamper our understanding of brain morphology among the crownward-most representatives of the avian stem group. Interrogating the neuroanatomical features of early euornitheans therefore constitutes a top priority for future work; such efforts, and the continued discovery of exceptionally preserved, three-dimensional cranial remains of Mesozoic avialans, will be needed to clarify the phylogenetic and temporal origins of a morphologically modern avian brain.

### (b) Palate morphology: origin of a kinetic beak

Most crown birds exhibit cranial kinesis [54], the capacity to move parts of the rostrum independently from the braincase. This specialization enhances dexterity and precision of the bill—faculties that became all the more important as the ancestors of birds sacrificed hands capable of fine manipulation in favour of wings as avian flight arose [52,55]. While the kinetic capabilities of extant bird skulls continue to be documented in greater detail [56], these capabilities are facilitated in part by specialized joints within the palate, with recent discoveries clarifying the pattern and timing by which this increased capacity for palatal mobility arose. In the oldest and most stemward avialan known, *Archaeopteryx*, the palate would have remained plesiomorphically rigid, with the organization of the palate’s components (paired vomers, palatines, pterygoids and ectopterygoids) resembling that of non-avian theropod dinosaurs (figure 1*b*) [57–59]. The vomers of *Archaeopteryx* are narrow and elongate, and contact the caudally positioned pterygoids via posteriorly directed rami [57,60,61]. The pterygoids are extremely long rostrocaudally, spanning almost the entire length of the palate, and contact the quadrates caudally [57,60,61]. Lateral to the rostral portion of the pterygoids, the palatines are slender and rostrocaudally elongate, contacting the pterygoids posteriorly [57,60,61]. Tongue-shaped wings of the ectopterygoids [61] extend laterally from the pterygoid, acting as a bridge to the jugal [62]. Although palate preservation is rare among stemward avialans other than *Archaeopteryx*, recent insight from *Jeholornis* [63] suggests other early avialans likely retained a generally similar palatal arrangement.

This plesiomorphic palatal arrangement did not undergo substantial modification among early pygostylians, as revealed by insights from *Sapeornis* [62,64]. It is currently unclear whether this was also the case among confuciusornithids, whose edentulous beaks are convergent with those of crown birds (e.g. [65–67]). Despite an abundant Early Cretaceous fossil record, the confuciusornithid palate remains poorly known as a result of a dearth of complete, three-dimensionally preserved cranial remains [65,68]. Among Enantiornithes, only a few taxa spanning the Early to Late Cretaceous (e.g. *Chiappeavis* [69], *Gobipteryx* [70], *Yuornis* [71], *Yuanchuavis* [72] and *Navaornis* [36]) preserve adequate palate material to inform discussions of broad patterns of avialan palate evolution. While isolated enantiornithine remains exhibit broadly similar palatal architecture to *Archaeopteryx*, most remains are too scanty to confirm whether the enantiornithine palate as a whole retained such a plesiomorphic condition.

Among the greatest outstanding questions surrounding enantiornithine palate morphology regards the uncertain presence of ectopterygoids. These large, rounded elements buttress the palate against the lateral wall of the skull in non-avian theropods and early avialans such as *Archaeopteryx* and *Sapeornis* [62], preventing motion of the palate, but their presence in Enantiornithes is controversial. While ectopterygoids were originally interpreted as present in *Gobipteryx* [73], this identification has been disputed [35], and subsequent descriptions have not unequivocally identified enantiornithine ectopterygoids despite the vast number of specimens discovered to date [74]. *Falcatakely*, a possible enantiornithine known only from a bizarre, isolated skull from the latest Cretaceous of Madagascar, exhibits unmistakeable large ectopterygoids [75], yet the referral of *Falcatakely* to Enantiornithes is not foregone [76], and certain phylogenetic analyses have recovered it outside Ornithothoraces altogether [77]. The sheer diversity and cranial disparity of enantiornithines may drive a tendency for isolated cranial remains—even those of non-avialan fossil vertebrates—to be drawn towards Enantiornithes in phylogenetic analyses [78], suggesting that uncertainty regarding both the phylogenetic position of *Falcatakely* and the presence of ectopterygoids in Enantiornithes will persist until discoveries of more complete fossil material emerge. Among early representatives of Euornithes, palates of specimens from the Early Cretaceous are also largely unknown. For several key taxa the quality of preservation is often poor, with palate elements sometimes present but usually not fully described [79–81].

Important palatal innovations appear within the crownward euornithine subclade Ornithurae, as illustrated by representatives of the clades Ichthyornithes and Hesperornithes [37,39,82,83]. Unlike stemward Mesozoic avialans, these taxa show no evidence of ectopterygoids, yet they exhibit palatal elements unknown in comparatively stemward taxa: paired hemipterygoids [37,39,82]. These articulate with the palatines rostrally and the pterygoids caudally [39,82], potentially providing an additional degree of freedom for kinesis within the palate. Although the palate of Hesperornithes is considered to be highly autapomorphic [39], that of Ichthyornithes may more closely resemble the plesiomorphic condition for crown birds, and documents changes in the articular relationships among the elements of the palate [37].

The distinction between the two major extant neornithine subclades (Palaeognathae and Neognathae; figure 1*b*) has long been supported by differences in their palatal morphology [35,84,85]. While neognaths exhibit a mobile joint between pterygoid and palatine enhancing the capacity for cranial kinesis, the palates of palaeognaths are more robust, with a rigid, often fused connection between the pterygoid and palatine [84–86]. Long thought to represent a neognath synapomorphy, the embryonic pterygoid generally undergoes a process of division, termed ‘segmentation’, in early ontogeny [87,88], resulting in the generation of a transient independent hemipterygoid positioned between the palatine and pterygoid in immature specimens close to hatching [82,87,89–96]. Unlike the condition in Ichthyornithes and Hesperornithes, in adult neognaths the hemipterygoid fuses with the caudal end of the palatine such that it ceases to be identifiable as an independent element [39,94,96].

In contrast to neognaths, the pterygoids of palaeognaths do not undergo segmentation, resulting in a palate conformation broadly similar to that of non-avian theropod dinosaurs and stemward Mesozoic avialans. As such, the palate morphology of palaeognaths was long assumed to retain the plesiomorphic condition inherited from the last common ancestor of crown birds [97–99]. However, the discovery of a well-preserved pterygoid in the ichthyornithine *Janavis* [37], coupled with the well-preserved palatines and hemipterygoids recently described from *Ichthyornis* [39], suggests that Ichthyornithes exhibited palates morphologically, and presumably functionally, similar to those of extant chickens (Galliformes) and ducks (Anseriformes), which have kinetic palates. These findings suggest that crownward stem birds, and presumably the earliest crown birds themselves, possessed mobile, neognath-like palates, unlike those of extant palaeognaths.

Deeper insight into avialan palatal evolution is essential for understanding the origin and refinement of avian cranial kinesis [62]. The loss of a solid jugal–postorbital bar and ectopterygoids were necessary for freeing the upper jaw to move relative to the braincase, although poor fossil preservation continues to obscure the precise timing of these transitions in Mesozoic avialan evolutionary history [62,88]. Indeed, the propensity for many Mesozoic avialan fossils to be preserved flattened [35,45,100] ensures that numerous questions regarding the Mesozoic evolutionary history of the avialan palate currently remain unanswered [37,39,62].

### (c) Pectoral girdle: a shoulder ready for take-off

The evolution of powered flight drove numerous transformations of the avian skeleton [16,101–106]. This is illustrated most clearly by the flight apparatus, which includes the pectoral girdle and forelimbs. Although much remains unknown regarding how and when maniraptoran dinosaurs first took to the air [11,107,108], recent work has helped clarify our understanding of the origin of a morphologically modern avian flight apparatus. Forelimb morphology was substantially transformed as the dexterous arms of ancestral maniraptorans became the highly specialized wings of avialans [77,109,110], and the pectoral girdle simultaneously underwent dramatic transformations linked to the evolution and refinement of powered flight [11,110–115].

**Figure 2.**
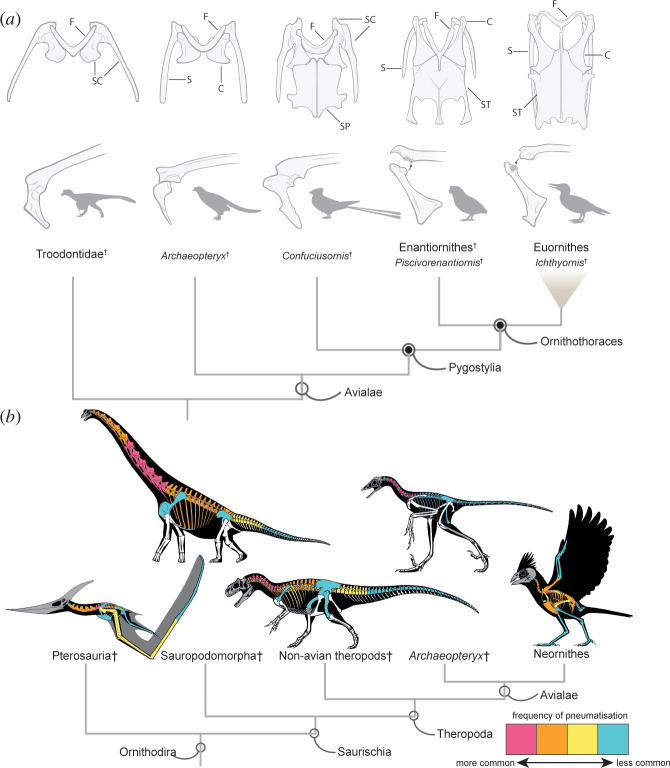
Evolution of the avian pectoral girdle and PSP. (*a*) Top: articulated paravian pectoral girdles in ventral view. Bottom: close-up of the glenoid region of the shoulder joint. Scapula and coracoid are fused into a scapulocoracoid in adult Troodontidae, *Archaeopteryx* and *Confuciusornis*, and unfused in Ornithothoraces (except ratites). Arrows between coracoid and scapula indicate divergent mechanisms for linking these elements in Enantiornithes and Euornithes. Abbreviations: C, coracoid; F, furcula; S, scapula; SC, scapulocoracoid; SP, sternal plates (unfused); ST, sternum. Illustrations modified from [77,111,116,117]. (*b*) Phylogeny of Ornithodira; colours indicate approximate frequency of pneumatization across the skeleton reported within each clade.

The pectoral girdle consists of the sternum, paired coracoids and scapulae, and the furcula or wishbone. This morphological unit connects the forelimbs to the trunk, provides the primary attachment surfaces for flight muscles and guides the tendons connecting the flight muscles to the wings. The pectoral morphology of stemward avialans such as *Archaeopteryx* does not differ much from that of non-avialan paravian dinosaurs, exhibiting simple, roughly quadrangular coracoids fused to the scapulae, a short and robust boomerang-shaped furcula, and no evidence of an ossified sternum (figure 2) [61,111,116,118–122]. Sternal remains are also unknown from the comparatively crownward avialan *Sapeornis*, suggesting that this element was either absent or largely cartilaginous in certain early avialans, hindering its fossilization potential [111,123,124]. Among stemward avialans, Jeholornithiformes and non-ornithothoracine pygostylians exhibit proportionally larger and more elongate coracoids, elongate scapulae and ossified sterna composed of two incompletely fused sternal plates [111,116,125,126]. Notably, the pygostylian subclades Confuciusornithiformes and Jinguofortisidae convergently evolved fused scapulocoracoids (figure 2*a*), similar to the condition in many non-avialan theropods and extant flightless palaeognaths, despite otherwise exhibiting clear flight adaptations [65,127,128]. It is unclear to what degree the comparatively plesiomorphic morphology of the pectoral girdle of non-ornithothoracine avialans impacted their flight capabilities, but it has been suggested that sustained flapping and highly manoeuvrable flight would have been impossible [110,115,129].

The evolution of more advanced flight specializations within Ornithothoraces was accompanied by the evolutionary acquisition of more complex pectoral morphologies in Enantiornithes and Euornithes [110,111,116,130,131]. These major clades share elongate coracoids, a complex coracoid–scapula articulation [111], a robust acromion process of the scapula [132], more acutely angled furculae [133] and fully fused sterna, in most cases exhibiting a sternal keel serving as the attachment point for the main flight muscles; these muscles were likely anchored to the coracoid and furcula in non-ornithothoracine avialans instead [110,111,116,123,134]. However, the pectoral morphologies of Enantiornithes and Euornithes differ in important ways, including divergent mechanisms for linking the coracoid and scapula: in Enantiornithes, the coracoid exhibits a convex protrusion inserting into a concave facet on the scapula [111,135–138], whereas the opposite relationship exists in Euornithes (including crown birds), where a cotyle on the coracoid receives a condyle from the scapula (figure 2*a*) [116,139]. Furthermore, recent work has illustrated that this mechanism represents the only point of articulation between the scapula and coracoid in Enantiornithes, whereas in Euornithes, as in all other avialans, the scapula exhibits an additional elongate point of contact with the coracoid [111]. The coracoids of most Euornithes (and only a few Enantiornithes; [140]) exhibit a procoracoid process directing flight muscle tendons arising on the sternum to the humerus [116,141,142]. In most crownward Euornithes and in crown birds, the procoracoid process contacts the scapula and the furcula (although variability exists) [139,143], fully enclosing these tendons in a so-called ‘triosseal canal’, the origin of which may have accompanied a transition to more active flapping flight and more sophisticated take-off capabilities [11,77,110,144]. The presence of an incomplete or incipient triosseal canal in Enantiornithes and stemward Euornithes is contentious [111,116,132], but it is likely that Enantiornithes exhibited a tendon arrangement distinct from that of living birds [110,111]. Most enantiornithines exhibit broad sterna that are mediolaterally wider than rostrocaudally long; their sternal keels–when present–are short, rounded and extend only along the caudal portion of the sternum (figure 2*a*) [145,146]. In most Euornithes, in contrast, the sternum is longer than wide, with a well-developed sternal keel that extends along the entire rostrocaudal axis of the bone and, in crownward ornithurines and many living birds, beyond its rostral margin (figure 2*a*) [77,131,147]. This more extensive keel has been suggested to be related to the displacement and expansion of the attachment points for the flight muscles related to take-off from the ground [110]. This inference is of particular importance for non-neornithine Euornithes, which have been interpreted as being predominantly ground-dwelling or semiaquatic [24,130], contrasting with the primarily arboreal ecologies inferred for Enantiornithes [110,148]. Notably, Avisauridae, a clade of Late Cretaceous enantiornithines, convergently evolved a similarly well-developed sternal keel, suggesting a similar muscular rearrangement to that of Euornithes [149–151]. Contrasting morphologies in Enantiornithes and Euornithes are also evident in the furcula: in the former, the furcula is V-shaped with a narrow, long and rod-like hypocleideum [152]; in the latter, the furcular symphysis is more rounded and a hypocleideum is generally absent [118].

Beyond osteological evidence for the evolution of powered flight, important insights into the nature of early bird flight can be gleaned from exceptionally preserved remains of fossil avialans. These can preserve soft tissues of the wing and pectoral apparatus, including feathers and propatagia [45,153–155], with implications for understanding the evolution and integrated function of the flight apparatus. Although the wing plumage of early avialans appears superficially similar to that of crown group birds (with the potential exception of Anchiornithidae, which may exhibit evidence of secondary flight reduction [156]), the feather structure of non-ornithothoracine avialans seems comparatively poorly suited for powered flapping flight [129]. Ornithothoraces, in contrast, possessed advanced crown bird-like flight feathers and alulae, which likely conferred sophisticated flight potential comparable with that of their living relatives [107,129]. Inferences related to the flight style of several fossil avialans have been made based on skeletal morphology and exceptionally preserved soft tissues, suggesting a wide diversity of flight styles arising early in bird evolutionary history [152,153,157–161], including thermal soaring [162] and bounding flight [163,164]. However, such inferences are limited by a poor understanding of the biomechanics of the flight apparatus in all but the crownward-most known stem birds [111,117]. This is largely due to the continuing scarcity of complete and articulated three-dimensional avialan remains, complicating the application of modern biomechanical methods to address these questions [107]. Nonetheless, the continued development of approaches for fossil retrodeformation (e.g. [117]), and computational biomechanical analysis (e.g. [165]), are poised to cast light on these fascinating questions in coming years.

### (d) Skeletal pneumaticity: lightening the load

Birds are the only extant tetrapod group to exhibit PSP; that is, the invasion of the respiratory system into elements of the postcranial skeleton, resulting in the replacement of comparatively dense marrow with air-filled cavities lined with epithelia [166–169]. PSP is an important avian specialization that lightens the skeleton, exhibited by most living birds except some aquatic divers [170], making it one of the most distinctive aspects of avian skeletal morphology.

The evolutionary origins of avian PSP may trace back as far as the Triassic Period (approx. 250–210 Ma), as pneumatic postcranial skeletal elements are evident in pterosaurs [171–174], sauropodomorph dinosaurs [175–179] and both avialan and non-avialan theropod dinosaurs [37,125,170,180,181]. The potential for skeletal pneumaticity to alter the relationship between body mass and body volume [170] has played a clear role in the evolution of enormous body size in the largest terrestrial and volant animals to have ever lived (sauropods and pterosaurs, respectively). Among crown birds, body mass has only been weakly correlated with the extent of PSP [166,170,182,183]. Rather, the relaxation of constraints on PSP development in crown birds has likely allowed for frequent adaptive decoupling of body mass–volume relationships, resulting in PSP patterns driven by selection for particular ecologies and locomotor strategies [166,170,183–186].

Despite the broad phylogenetic distribution of PSP across Ornithodira (figure 2*b*), its absence in all known ornithischian dinosaurs and many early diverging saurischians supports a hypothesis that an invasive air sac system evolved multiple times—once in the lineage leading to pterosaurs, once in sauropods and another—most likely homologous with that of birds—in theropods [187]. As such, the current consensus suggests that PSP was absent in the last common ancestor of Ornithodira, and even Saurischia, though the pulmonary tissues underpinning the capacity to evolve an invasive pneumatic system are thought to be homologous across Ornithodira [167,187,188]. All three Mesozoic ornithodiran groups exhibiting PSP generally show a pattern where pneumatization of the cervical and anterior dorsal/thoracic vertebrae precedes the evolution of more extensive skeletal pneumatization (figure 2*b*) [170–172,178,180,189].

Within Avialae, *Archaeopteryx* shows clear evidence of axial pneumaticity of the cervical and anterior thoracic vertebrae and likely some ribs, but ambiguous evidence of PSP in the appendicular skeleton (figure 2*b*) [180,190]. This pattern of PSP appears to be no more expansive than the common pattern observed among non-avian theropods [180,191]. The evolution of PSP is poorly understood among more crownward stem birds, due to the difficulties in unambiguously assessing evidence for PSP in specimens that are taphonomically flattened, crushed and relatively small compared with most other fossil bird-line archosaurs. However, pneumaticity of portions of the vertebral column (e.g. in Confuciusornithiformes; [65]), as well as expansions into ribs and sterna (e.g. in Ichthyornithes; [37,77]) have occasionally been reported in both early and near-crown avialans.

In contrast to axial pneumaticity, there are currently only two instances of pneumatic forelimb elements (humeri and ulnae) reported in non-crown Avialae, both belonging to Late Cretaceous Enantiornithes from Argentina [137,151]. The presence of pneumatic foramina in these enantiornithines, coupled with their apparent absence in all other avialans outside crown group birds (seemingly including even highly pneumatized crownward ornithurans [37]), suggests the plesiomorphic pattern of postcranial pneumatization observed in non-avian theropods may have been retained throughout almost the entirety of non-crown avialan evolutionary history. This scenario hints at the possibility of independent evolutionary acquisitions of expansive PSP in Enantiornithes and crown birds; however, our understanding will be refined as additional non-neornithine avialans are investigated in detail.

Unlike most extinct ornithodirans, extant birds exhibit extremely variable degrees of postcranial pneumaticity, both in terms of the proportion of pneumatized elements across the skeleton [166,170,183,184], and in the extent to which individual postcranial skeletal elements are filled with air [185,192]. When present, the most common pattern of postcranial skeletal pneumatization among crown birds involves a few vertebrae at the cervicothoracic border [170], reminiscent of the ancestral pattern apparent in their extinct ornithodiran relatives. However, elaboration of this pattern is extremely widespread among crown birds, with pneumatic diverticula often expanding into much of the vertebral column, ribs, sternum, pectoral and pelvic girdle elements, humeri and femora [166,170]. Pneumatization of the distal limb elements is comparatively rare, though this occurs in taxa with especially pneumatic skeletons (e.g. pelicans; [170]). At the other extreme, some taxa exhibit completely apneumatic postcranial skeletons (e.g. penguins and loons), though this pattern of PSP reduction is generally associated with specialized aquatic diving ecologies that impose selective constraints against the buoyancy conferred by PSP [170,183]. The wide range of variation in PSP among crown birds indicates a relaxation of constraints associated with the development of skeletal pneumaticity with respect to the plesiomorphic theropod condition, in which pneumaticity is generally restricted to the axial skeleton.

## 2. Conclusions

In only a few decades, the field of avian palaeobiology has progressed dramatically. A rich picture of Mesozoic avialan diversity and interrelationships has emerged, providing the necessary phylogenetic framework for discerning the evolutionary patterns and processes that gave rise to many of the distinctive characteristics of extant birds. Investigations like those covered in the present review have fundamentally reshaped our understanding of how numerous aspects of avian biology evolved.

While flattened and/or incomplete fossil remains have formed the basis for many of the most exciting Mesozoic avialan discoveries to date, two-dimensional fossils are limited in their ability to answer many questions about avian morphology. Indeed, major recent advances in our understanding of the character systems covered in this review have depended on the discovery of exceptional, though rare, three-dimensionally preserved fossils. As additional undistorted specimens emerge to fill key phylogenetic gaps within Avialae, and advances in three-dimensional fossil imaging continue to progress, our understanding of the origins of the ‘modern’ avian condition of innumerable morphological and functional features will continue to be refined.

**Figure 3.**
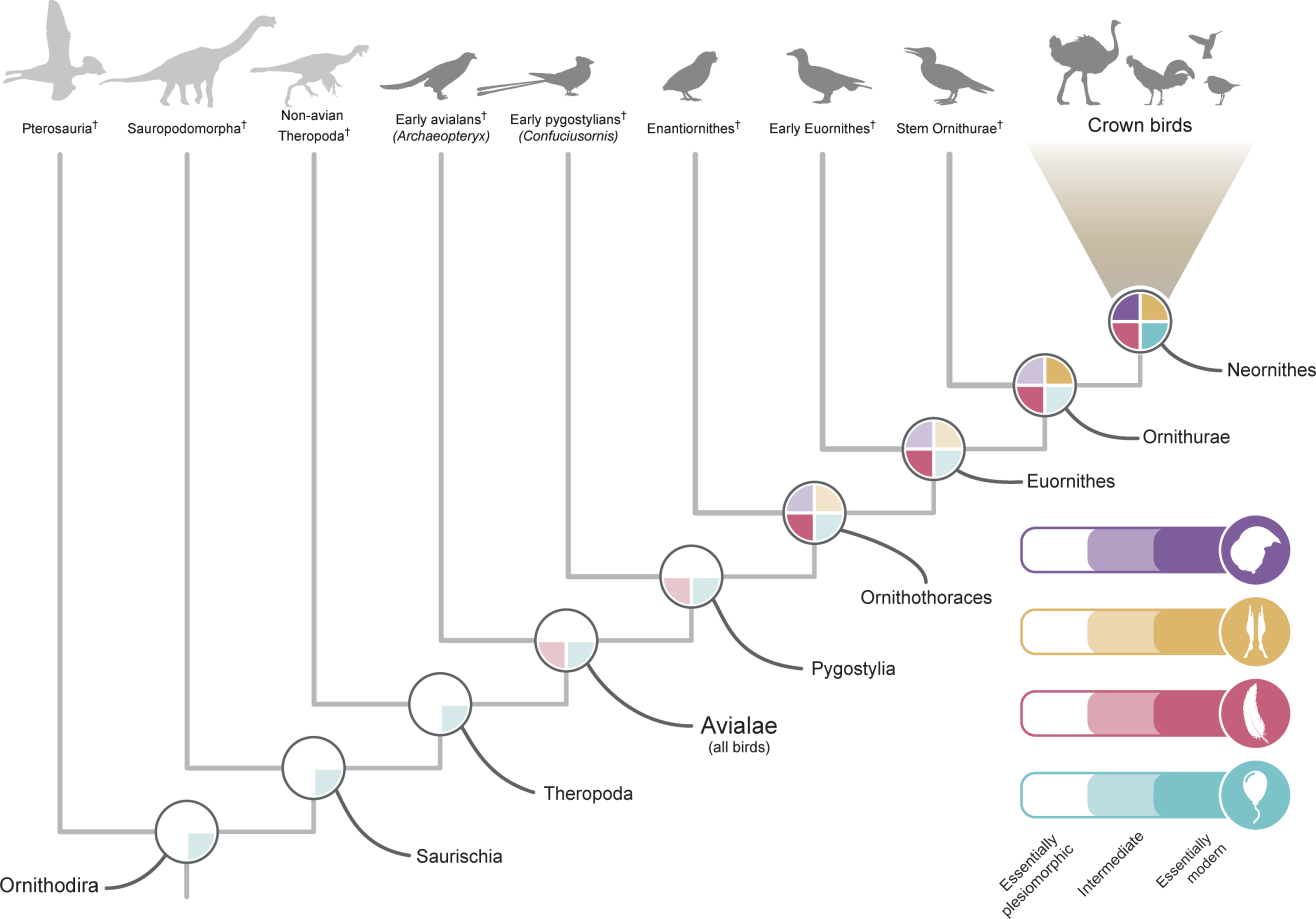
Phylogeny of bird-line archosaurs and the hierarchical evolutionary acquisition of a bird-like phenotype. White sectors of pie charts indicate retention of the plesiomorphic, non-neornithine condition. Faintly coloured sectors indicate an intermediate, near-crown-bird-like condition and saturated colours indicate the condition observed in crown birds. Purple, brain morphology; yellow, palate morphology; red, pectoral girdle morphology; blue, PSP.

Additionally, although *Archaeopteryx* remains the stratigraphically oldest and phylogenetically most stemward avialan known, as well as the first to be discovered, future progress towards understanding the origin of the crown bird body plan will depend far more on filling as-yet undersampled phylogenetic intervals within Avialae than on interrogations of *Archaeopteryx* alone. Such crucial, yet poorly sampled intervals include the early stages of evolution within Ornithothoraces, the early stem lineages of both Palaeognathae and Neognathae, and the crownward-most portion of the avian stem group (crownward of Hesperornithes). Indeed, when the origin of the crown bird condition across different anatomical systems is depicted phylogenetically (figure 3), late-stage refinements in all character systems tackled in the present review are ubiquitous along the crownward-most portion of the avian stem. Important evolutionary transformations must have taken place somewhere along the as-yet unsampled interval crownward of Hesperornithes, prior to the origin of crown birds themselves. New insights into this region of avialan phylogeny are poised to dramatically improve our understanding of how and when the distinctive biological characteristics of the world’s most diverse terrestrial vertebrates arose.

## Data Availability

This article has no additional data.
